# Development of a novel knockout model of retinitis pigmentosa using *Pde6b*-knockout Long–Evans rats

**DOI:** 10.3389/fmed.2022.909182

**Published:** 2022-09-21

**Authors:** Jee Myung Yang, Bora Kim, Jiehoon Kwak, Min Kyung Lee, Jeong Hoon Kim, In-Jeoung Baek, Young Hoon Sung, Joo Yong Lee

**Affiliations:** ^1^Department of Ophthalmology, Asan Medical Center, University of Ulsan College of Medicine, Seoul, South Korea; ^2^Department of Ophthalmology, Dongguk University Ilsan Hospital, Goyang, South Korea; ^3^Asan Institute for Life Sciences, Asan Medical Center, Seoul, South Korea; ^4^Department of Convergence Medicine, University of Ulsan College of Medicine, Seoul, South Korea

**Keywords:** *Pde6b*, Long–Evans rats, retinitis pigmentosa, bone spicule, retinal degeneration, electroretinogram

## Abstract

Although rats with melanin-pigmentated retinal pigment epithelial (RPE) cells are physiologically more appropriate models for human eye research than their albino counterparts, reliable models from the former strain are not available to study retinal degeneration. Here, we describe the development of a novel *Pde6b*-knockout Long–Evans (LE *Pde6b* KO) rat model that recapitulates key features of human retinitis pigmentosa (RP). After the generation of the *Pde6b*-knockout Sprague–Dawley rats with the CRISPR-Cpf1 system, the LE rat was back-crossed over 5 generations to develop the pigmented LE *Pde6b* KO strain. Interestingly, LE *Pde6b* KO displayed well-developed bone-spicule pigmentation; a hallmark of fundus in patients with RP which cannot be observed in non-pigmented albino rats. Moreover, the rat model showed progressive thinning of the retina, which was evident by intravital imaging with optical coherence tomography. Histologically, significant atrophy was observed in the outer nuclear layer. Functionally, LE *Pde6b* KO presented a marked decrease of amplitude level during electroretinogram testing, demonstrating significant loss of visual function. Therefore, these findings suggest that the LE *Pde6b* KO model robustly recapitulates the hallmark phenotype of RP. We believe that the LE *Pde6b* KO model may be used effectively for preclinical translational research to further study retinal degeneration.

## Introduction

Retinal degenerative disorders, such as retinitis pigmentosa (RP) and age-related macular degeneration (AMD), are major causes of blindness worldwide (1, 2). Although previous research has focused on restoring vision by replacing photoreceptors and retinal pigment epithelial (RPE) cells, long-term results are still lacking owing to the neurodegenerative nature of these diseases (3–6). Moreover, current clinical treatments that are targeted at delaying disease progression have had limited impact; thus, the need for preclinical studies that can overcome this issue is considerable (5, 7).

Rats are widely used for retinal research; however, different strains have varying physiological functions and pathological processes, and using the appropriate strain is critical for modeling human diseases (8). Sprague–Dawley (SD) rats are one of the most extensively used outbred laboratory rat populations. We have previously utilized the SD strain for studying retinal degenerative disorders; however, their non-pigmented albino feature limits the understanding of the role of melanin pigment as an essential component in the RPE that acts as a photo-screen and anti-oxidant (9). Although the non-pigmented SD rat is ideal for observing changes in pigmented RPE cells after transplantation, because of the lack of the antioxidant function and photo-screening against visible light and ultraviolet radiation, SD rats cannot fully simulate human retinal neurodegeneration, thereby necessitating the development of a melanin-pigmented rat strain.

Long–Evans (LE) rats possess pigmentation in the RPE layer and display better visual function (10). Therefore, we hypothesized that LE rats with pigmented RPE are a physiologically more appropriate model for human eye research than SD rats. Here, we developed and explored the *Pde6b*-knockout LE rat model that may enhance the utility of these animal models in preclinical research on retinal degeneration.

## Materials and methods

### Animals

**FIGURE 1:**
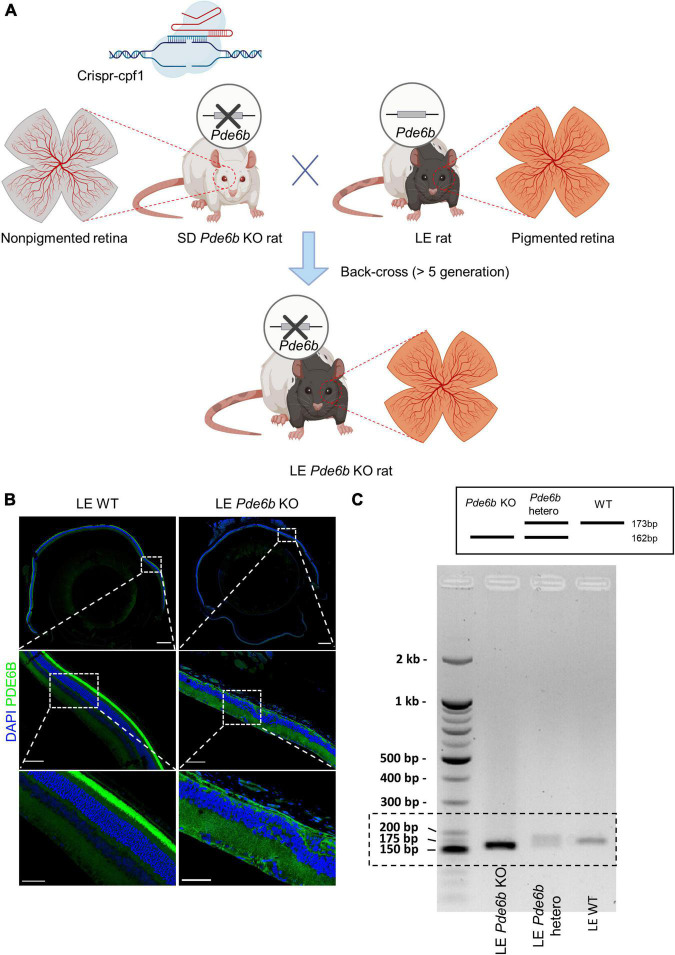
Generation of pigmented *Pde6b*-knockout Long–Evans rats. **(A)** Schematic illustration depicting the process of generating the Long–Evans *Pde6b*-knockout rat (LE *Pde6b* KO). A CRISPR-Cpf1 engineered Sprague–Dawley (SD) rat with a deletion of the *Pde6b* gene is back-crossed over at least five generations with LE rats. Schematic created with BioRender. **(B)** Immunofluorescent images of the cross-sectioned retina of 17-weeks old rat. Insets indicate the location of the magnified images. Scale bars, 500 μm (top), 100 μm (middle), 50 μm (bottom). **(C)** Genotyping result of the WT, *Pde6b*-heterozygote, and *Pde6b*-knockout rat of the Long-Evans strain for the detection of the mutant allele. WT, wild-type.

All experimental procedures were conducted in accordance with the statement for the use of animals in ophthalmic and vision research provided by the Association for Research in Vision and Ophthalmology. All experimental SD and LE rats (OrientBio, Gyeonggi, Korea) were housed in a specific pathogen-free animal facility at the Asan Institute for Life Sciences, in the Asan Medical Center. *Pde6b*-knockout SD rats were generated as previously described (11), and then back-crossed with LE rats over at least five generations (Figure 1A). Rats were anesthetized by intramuscularly injecting a mixture of tiletamine hydrochloride, zolazepam hydrochloride (Zoletil, 0.4 mL/kg; Virbac Laboratories, Carros, France), and xylazine (Rompun, 0.6 mL/kg; Bayer Korea Ltd., Seoul, Korea). All animal care and experimental methods were reviewed and performed in accordance with the institutional animal care committee’s authorized protocol (No. 2020-02-151) at the Asan Institute for Life Science. Each experiment was performed by utilizing tissues from at least three animals for statistical validation of the results.

### Fundus photography and optical coherence tomography

After anesthesia, the pupils were dilated with Mydrin-P (0.5 percent tropicamide and 0.5% phenylephrine hydrochloride; Santen Pharmaceuticals, Osaka, Japan) for intravital retinal imaging. A Hycell physiological solution (2% hydroxypropylmethylcellulose; Samil Pharmaceuticals, Korea) was applied to the cornea, and refraction was equalized using a microscope coverslip. Fundus and optical coherence tomography (OCT) imaging (OCT; IIS Science) were used to observe clinical signs of degeneration of the retina (6).

### Electroretinogram

Electroretinogram (ERG) testing was performed as previously described (6, 12). The rats were dark-adapted for at least 12 h prior to the recording. During the measurement of the scotopic and photopic reactions, only dim red light was employed. All ERG measurements were obtained using a Phoenix Micron IV system (Phoenix Research Labs, Pleasanton, California, USA) and processed with LabScribeERG software (v. 3; Phoenix Research Labs). Pupils of the rats were dilated after anesthesia, and corneas were identified using a gold-plated objective lens. For measurement, needle electrodes were inserted in the forehead (reference) and tail (ground). After the recording, infrared light was utilized to keep the animals warm until they recovered consciousness.

### Histologic analysis

The eyes were fixed overnight with 4% paraformaldehyde (PFA) and embedded in paraffin to be cut into 4-μm sections for H&E staining. For those cases with cryosectioned retinas, the eyes were enucleated and fixed overnight with 4% PFA and underwent gradient dehydration with sucrose. Subsequently, the processed eyes were snap-frozen in optimal cutting temperature compound. The retinas were blocked in PBST (0.5% Triton X-100 in PBS) and treated overnight with 5% normal goat serum with an anti-PDE6B antibody (sc-377486; Santa Cruz Biotechnology, 1:100 dilution) at 4°C. Following a wash in 0.5% PBST, the samples were incubated for 4 h at room temperature with species-specific Alexa Fluor-coupled secondary antibodies. To identify the nucleus, DAPI/Hoechst dyes were utilized. The samples were then washed in 0.5% PBST at least five times and mounted with a mounting medium (Vectashield, Vector Laboratories, Burlingame, CA, USA). A Zeiss LSM 780 confocal microscope (Carl Zeiss, Berlin, Germany) was used to obtain immunofluorescence data.

### Genotyping

Rat genotyping was conducted using genomic DNA extracted from tissues obtained from tail biopsies. To detect the deletion of *Pde6b* with a frameshift mutation, a primer pair (forward primer 5′-ATGGGAACCCCACCTTTGCC-3′ and reverse primer 5′-GACGCTCTCTTGCATGTCCT-3′) producing a short PCR product (173 bp in the wild-type mouse) was employed as previously described (11).

### Statistics

R v. 4.1.2 (R Foundation for Statistical Computing, Vienna, Austria) and GraphPad Prism v. 7.0 (GraphPad Software, San Diego, CA, USA) were used for statistical analysis. Data are shown as mean ± standard deviation (SD). The Shapiro–Wilk test was used to test normal distribution. The Mann–Whitney U test was used to compare non-normally distributed metrics between the groups, and the student’s *t*-test was used to compare normally distributed metrics. A *P* value < 0.05 was considered statistically significant.

## Results

### Generation of *Pde6b*-knockout Long–Evans rats

Initially, *Pde6b KO* rats from the SD strain were generated with CRISPR-Cpf1 technology as previously described (11). After successful generation, the *Pde6b KO* SD rats were crossed with LE rats and then back-crossed for at least 5 generations to generate a novel LE *Pde6b* KO rat (Figure 1A). The deficiency of the PDE6B protein in the LE *Pde6b KO* rats was confirmed by immunostaining of the retina, which showed deficient immunofluorescence at the photoreceptor level (Figure 1B). Additionally, defect of the *Pde6b* gene was confirmed by PCR genotyping (Figure 1C).

### *Pde6b*-knockout Long–Evans rats recapitulate bone-spicule pigmentation, a typical characteristic of human retinitis pigmentosa

**FIGURE 2:**
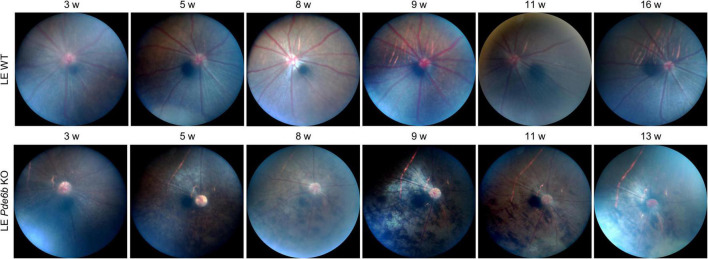
Fundus changes associated with bone-spicule pigmentation in the *Pde6b*-knockout Long–Evans rats. Serial fundus photography showing marked development of the bone-spicule pigmentation, which is a hallmark of retinitis pigmentosa. LE WT, wild-type Long–Evans; LE *Pde6b* KO, *Pde6b*-knockout Long–Evans rat.

To investigate the gross retinal phenotype of the LE *Pde6b* KO rat, we continuously examined fundus photography serially up to 4 months. Interestingly, diffuse bone-spicule pigmentation, one of the hallmarks of retinal degeneration in patients with RP (13), was observable as early as 3 to 5 weeks (Figure 2). Additionally, the optic nerve progressively became pallor, which was representative of degeneration of the optic nerve axons after retinal degeneration. Overall, the fundus phenotype of LE *Pde6b* KO rat robustly recapitulated the fundus of human RP.

### *Pde6b*-knockout Long–Evans rats demonstrate progressive retinal degeneration

**FIGURE 3:**
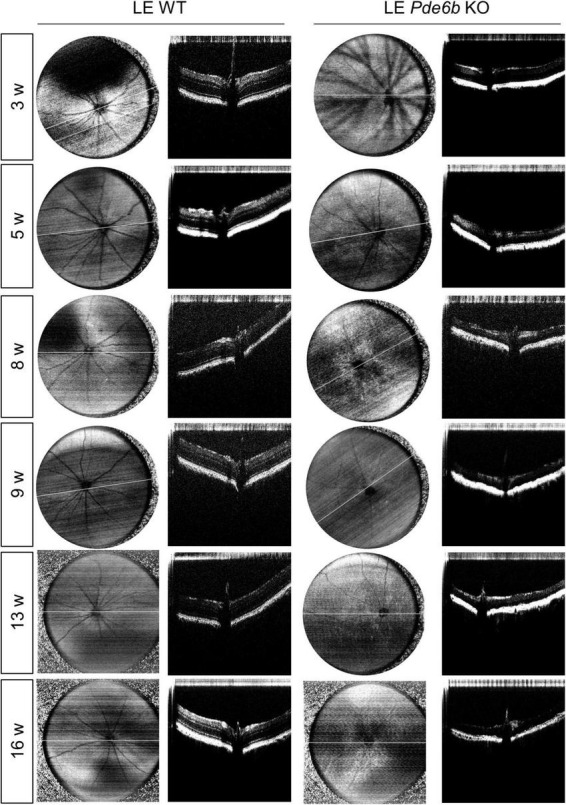
Serial infrared fundus images and optical coherence tomography (OCT) of the wild-type Long–Evans rats and the *Pde6b*-knockout Long–Evans rats. Representative infrared fundus images and OCT shows progressive thinning of the *Pde6b*-knockout (LE *Pde6b* KO) retina compared with wild-type Long–Evans rats (LE WT) rat over a period of 16 weeks.

**FIGURE 4:**
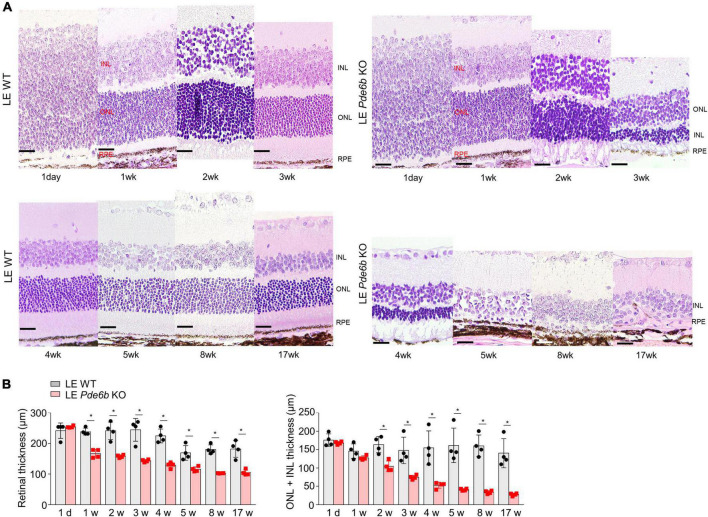
Histologic analysis of the retinas of wild-type Long–Evans rats and *Pde6b*-knockout Long–Evans rats. **(A)** Serial images of the H&E staining of cross-sectioned retinas of wild-type Long–Evans rats (LE WT) and *Pde6b*-knockout rats (LE *Pde6b* KO) at each time point over a 17-week period. Compared with LE WT rats, LE *Pde6b* KO rats showed progressive thinning of the total retinal thickness, especially in the outer nuclear layer. **(B)** Quantification of retinal thicknesses over 17 weeks. INL, inner nuclear layer; ONL, outer nuclear layer. Scale bars, 20 μm. * *P* < 0.05, *n* = 4 eyes per group.

For intravital analysis of the retinal structure, we performed OCT imaging of the LE *Pde6b* KO rats (Figure 3). Interestingly, this showed progressive thinning of the retinal layer in the LE *Pde6b* KO rats; the thickness was markedly lower than that in the LE WT rats. For microscopic analysis of the retinal layer, H&E staining of the cryosectioned retina was performed (Figure 4A). Notably, compared to the LE WT rat, LE *Pde6b* KO showed progressive thinning of the retinal layer, with significant atrophy of the outer nuclear layer which become hardly detectable at 5 to 8 weeks (Figure 4B). The inner nuclear layer was relatively well-preserved during observation.

### Functional analysis of *Pde6b*-knockout Long–Evans rats

**FIGURE 5:**
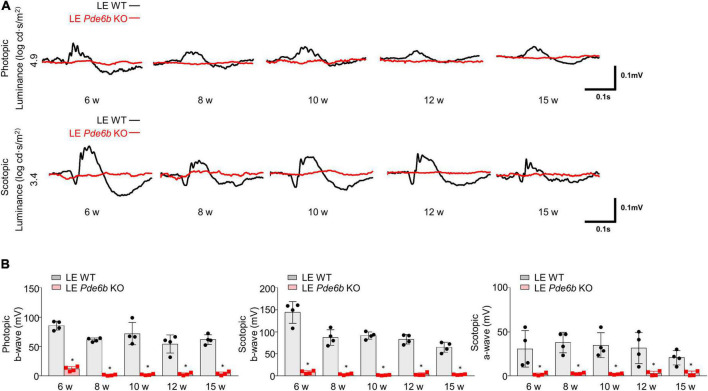
Electroretinogram (ERG) of *Pde6b*-knockout Long–Evans rats. **(A)** Representative ERG data showing significantly decreased amplitude in both photopic and scotopic response in the LE *Pde6b* KO rat at each time point. **(B)** Quantification of the amplitude of photopic b-wave, scotopic a- & b-wave. LE, Long–Evans * *P* < 0.05, *n* = 4 eyes per group.

Given that the functional decline of photoreceptors is a major cause of visual loss in patients with RP, we used an ERG to evaluate photoreceptor function (Figure 5). Comparable to the intravital and histology results, both photopic b-wave and scotopic b- & a-wave amplitudes were nearly flat from 6 weeks to 15 weeks. This finding implies that the LE *Pde6b* KO rat features significant functional impairment of both cone and rod photoreceptor cells.

## Discussion

In this study, we describe the basic phenotype of a novel LE *Pde6b* KO rat model simulating human retinal degeneration. Our findings showed that the rat model displayed bone-spicule pigmentation in the fundus, marked thinning of the retina (as detected by OCT), significant degeneration of the retinal neurons and photoreceptors, and functional impairment of cone and rod photoreceptors (as determined by ERG). These findings imply that our novel LE *Pde6b* KO rat robustly recapitulates the hallmark phenotypes of patients with RP and may provide a reliable model for translational research concerning human retinal degeneration.

The pigmented LE rat with its melanated RPE has a critical advantage over the commonly-used non-pigmented SD rat for retinal research. The RPE has a brown melanin granule, and its concentration varies in location of the retina (14). This melanin granule absorbs light and protects the eye from photo-oxidative stress, acting as a free radical stabilizer (9). With advancing age, the melanin content gradually decreases by lysosomal activity, thereby presenting less pigmentation in the aged fundus (14). Reportedly, there are significant differences in the visual perception of the aforementioned rat strains, with higher visual acuity in pigmented rat strains than that in albino rats (10). To this effect, the SD rat with its non-melanated RPE may not fully represent a normal healthy retina. Therefore, when inducing retinal degeneration by genetic modification, LE rats may be preferable over SD rats for functional testing of the retina compared to controls. Moreover, utilizing LE rats might be useful for evaluating the protective effect of certain therapeutic strategies on retinal function.

Notably, our rat model robustly displayed bone-spicule pigment configuration, which is one of the hallmarks of human RP. Histologically, bone-spicule pigmentation signifies translocation of the RPE subsequent to the loss of photoreceptor cells in patients with RP (15). This pigment-containing region is important for RP research as the area presents many pathologic changes in the RPE, blood vessels, glia, and neurons during RP pathogenesis. Although pigmentary degeneration is observed in some mouse models for retinal degeneration, such as the *rd10* mice (16), there is no reliable rat model, to our knowledge, that can consistently reproduce bone-spicule pigmentation in the fundus. Moreover, as the rat is more biologically parallel to humans, our rat model has greater translational value than previously published mouse models (17).

Another advantage of our rat model is the opportunity it provides to examine the function of the blood-retinal barrier (BRB) through fluorescein angiography (FAG). Without the presence of melanin pigment in the RPE, the background choroidal signal cannot be absorbed, making it difficult to differentiate between retinal vessels during FAG. Therefore, rats with pigmented RPE display better contrast between the retinal vessels compared to albino rats when FAG is performed (18, 19). With FAG, quantitative and qualitative evaluations of retinal leakage and edema can be observed to study cystoid macular edema, one of the major complications of RP (13). Interestingly, the RP retinal vessel shows the development of fenestrations in endothelial cells (15). These fenestrated vessels express a high level of PLVAP, one of the biomarkers of retinal hyper-permeability and inner BRB breakdown (20–22). As such, our LE *Pde6b* KO model can be utilized to investigate the pathophysiology of inner BRB breakdown in RP as well as to evaluate new therapies for the treatment of retinal edema in RP.

Existing rat RD models, such as the s334ter and P23H rat, accurately reproduce the degenerative process seen in human RP as shown by electrophysiologic data and ultrastructural histology (23–25). Although P23H and s334ter mutation of opsin gene is common in US, according to our NGS study, *Pde6b* is the most frequently affected gene in patients with RP in Asian population (26–28). Also, s334ter and P23H are RP models inherited in an autosomal dominant pattern, whereas our *Pde6b* KO model is an autosomal recessive pattern, showing differences in inheritance patterns. Although the phenotype may be similar, the difference in causative gene and genetic pattern of our model may provide useful tools for pathogenesis research and treatment development of RP. Furthermore, we found that RP patients with PDE6B variations presented earlier and received a diagnosis earlier than patients with RP brought on by other variants (26). Compared to existing rat models, our *Pde6b* KO rat model effectively reproduces the early pigmentary alterations seen in the fundus. In this respect, studying with our rat model may have an advantage of studying pigmentary degeneration which is readily visible intravitally through fundus imaging. Therefore, utilizing our rat model have advantage in developing strategies to prevent degeneration in subset of patients who bares *Pde6b* mutation.

Despite the advantages provided by our rat model, it does have a limitation; the duration for back-crossing the LE rat with the originally-developed SD *Pde6b* KO strain can be long. However, considering the significant expense and relatively low success rate for genetic manipulation, back crossing with an established animal model is a more effective and reliable method to create the new *Pde6b* KO model from the LE strain. Also, since our model is established, more can be used to produce the same genotype without previous back crossing. The low resolution of our animal OCT imaging tool is another limitation of our study since detailed analysis of the retinal layers to characterize degenerative changes of the photoreceptors layers was impossible (25).

In conclusion, we have developed a novel LE *Pde6b* KO rat model for studying retinal degenerative diseases, such as retinitis pigmentosa. Our rat model robustly recapitulates human retinal degeneration, especially with regards to bone-spicule pigmentation that is a characteristic phenotype in patients with RP. We believe our rat model may be a useful tool for translational research in the field of retinal degeneration.

## Data availability statement

The original contributions presented in this study are included in the article/supplementary material, further inquiries can be directed to the corresponding author.

## Ethics statement

The animal study was reviewed and approved by Asan Institute for Life Science.

## Author contributions

JL had full access to all of the data for the study and was principally involved in ensuring the integrity of the data and accuracy of the data analysis. JY, BK, JK, ML, JHK, I-JB, YS, and JL were responsible for designing and conducting the experiments. JY and JL drafted the manuscript. All authors contributed to the article and approved the submitted version.

## Abbreviations

AMD: age-related macular degeneration
BRB: blood-retinal barrier
ERG: electoretinogram
KO: knock-out
LE: Long-Evans
OCT: optical coherence tomography
PFA: paraformaldehyde
RP: retinitis pigmentosa
RPE: retinal pigment epithelial cell
SD: Sprague-Dawley.
